# Osmotic Demyelination Syndrome in a Patient With Tremors

**DOI:** 10.7759/cureus.30076

**Published:** 2022-10-08

**Authors:** Mona Ahmed, Peter Moffett

**Affiliations:** 1 Emergency Medicine, Virginia Commonwealth University (VCU) Health, Richmond, USA

**Keywords:** gait ataxia, hand tremor, horizontal nystagmus, mri imaging, electrolyte abnormalities, treatment of hyponatremia, osmotic demyelination

## Abstract

Osmotic demyelination syndrome is a neurological disorder caused by damage to the myelin sheath of brain cells secondary to rapid correction of hyponatremia. Clinical features are variable depending on the location of demyelination, with diagnosis confirmed by MRI. Once diagnosed, treatment is supportive. We present a 49-year-old female recently discharged from an outside hospital who presented with symptoms of tremors, ataxia, slurred speech, and confusion. The patient was diagnosed with osmotic demyelination syndrome based on the classic trident sign on MRI imaging. A review of her records showed rapid correction of serum sodium during her initial hospital visit.

## Introduction

Osmotic demyelination syndrome (ODS), now recognized as an umbrella term including central pontine myelinolysis and extrapontine myelinolysis, is a rare but preventable condition caused by rapid correction of hyponatremia. Changing osmotic forces compress fiber tracts, ultimately resulting in demyelination [1]. Clinical manifestations of ODS are highly variable and depend on the region of the brain affected, ranging from tremor or dysarthria to quadriparesis and locked-in syndrome [2]. Those at particular risk for the development of ODS include those with chronic alcohol use, chronic malnutrition, and liver transplant patients [3]. We present a case of ODS in a 49-year-old woman with a recent admission at an outside facility for alcohol withdrawal who presented with tremors, slurred speech, and confusion.

## Case presentation

A 49-year-old female presented to the emergency department with three days of tremors, loss of balance, slurred speech, and confusion. She had been discharged six days prior from another hospital after a stay for alcohol withdrawal. She had returned to that facility twice in the last week complaining of progressive loss of coordination and weakness. Her husband had reported needing to carry her around the house and brought her to our emergency department for evaluation. She denied ethanol consumption since her initial visit at the outside facility. She had not started any new medications, nor had she sustained any trauma. The patient did not have any history of liver disease from her alcohol use.

On exam, the patient had resting tremors of her bilateral upper extremities, horizontal nystagmus with lateral gaze, ataxia of the lower extremities with heel-to-shin testing, and occasional slurring of her speech. The rest of her physical exam was unremarkable.

**Figure 1:**
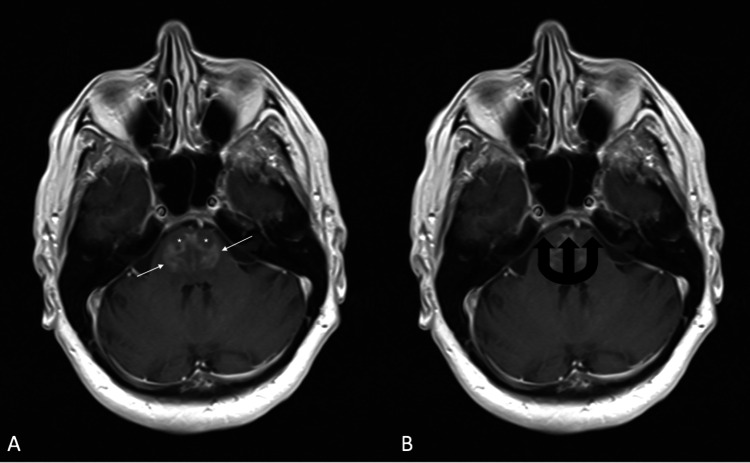
T1 Post Contrast MRI showing demyelination (A) of the pons sparing the corticospinal tracts (asterisks) and peripheral pontine fibers (arrows). This has been likened to a trident (B) or pig snout (not shown).

The patient was admitted, and an MRI was ordered. The MRI returned with T1 post-contrast views showing enhancement of the pons with sparing of the corticospinal tracts and peripheral pontine fibers (Figure 1). These imaging findings have been described as a “trident sign” or “pig snout” due to their characteristic shape [4].

The admitting physicians were able to obtain a report of “rapid sodium correction” during her prior hospital stay but did not obtain specific sodium values. Based on this report and the classic MRI findings, the patient was diagnosed with osmotic demyelination syndrome. During her inpatient stay, she was evaluated by neurology, physical therapy, occupational therapy, and the rehabilitation service and was discharged to an inpatient rehabilitation facility for supportive care. After intensive speech therapy and physical therapy, the patient was able to overcome her deficits and was left with intermittent neuropathic pain in her lower extremities.

## Discussion

Osmotic demyelination syndrome (ODS) is a rare condition caused by rapid correction of hyponatremia with compression of fiber tracts by changing osmotic forces. Patients at risk for the development of ODS are often chronically hyponatremic, giving the brain a chance to adapt to these conditions. When this hyponatremia is rapidly corrected, the brain is not protected from osmotic stress and there is a deficiency in organic osmolytes, resulting in cell shrinkage and demyelination [1]. The prevalence of ODS is approximately 0.25-0.5 percent in the general population [5].

Osmotic demyelination syndrome should be suspected in the presence of risk factors including electrolyte disturbances, malnutrition, and chronic alcohol use. Interestingly, manifestations of ODS are often delayed, presenting several days after overcorrection [6]. Diagnosis depends on obtaining a detailed medical history with a presentation of new neurologic symptoms noted after rapid correction of sodium levels as well as an MRI demonstrating demyelination sites. Demyelination occurs in the pons, cerebellum, lateral geniculate body, thalamus, and external capsules. Sites show hyperintensity in T2-weighted images and hypodensity in T1-weighted images [7]. Classic findings include hyperintense signals in the pons that spare the corticothalamic tracts and peripheral pontine fibers on T1 post-contrast imaging. This constellation of findings has been termed a trident or piglet sign due to its shape [4].

Slow correction of hyponatremia is the best way to prevent the development of this syndrome [8]. The optimal correction rate is much debated with some sources setting a maximum goal of 12 mmol/L over 24 hours and 25 mmol/L over 48 hours while others suggest lower rates in those at high risk, with a goal of 8 mmol/L in the first 24 hours [9]. Once the diagnosis has been established, treatment is largely supportive. No evidence-based treatments are available. Those left with extrapyramidal features have shown some response with dopamine agonists and other anti-dystonic medications [10]. Prevention is of utmost importance, as 33 to 55 percent of patients diagnosed with osmotic demyelination syndrome either die or remain permanently dependent on nursing care [7].

## Conclusions

Osmotic demyelination syndrome is a rare condition caused by rapid correction of hyponatremia and treatment is largely supportive once the diagnosis is established. Clinicians should be familiar with the risks, presentation, and prevention of ODS. Classic MRI findings such as the trident sign showing demyelination of the pons with sparing of the corticospinal tracts can aid in the diagnosis. Given that there is no evidence-based treatment, prevention is of the utmost importance.
